# Scaling an equity-oriented extended home visiting programme in Sweden: costs and cross-sectoral fiscal distribution

**DOI:** 10.1093/eurpub/ckag174

**Published:** 2026-09-25

**Authors:** Sergio Flores, Gunilla Lönnberg, Anna Sarkadi, Alice Brunnberg, Filipa Sampaio

**Affiliations:** Department of Public Health and Caring Sciences, Uppsala University, Uppsala, Sweden; Department of Public Health and Caring Sciences, Uppsala University, Uppsala, Sweden; Department of Public Health and Caring Sciences, Uppsala University, Uppsala, Sweden; Department of Public Health and Caring Sciences, Uppsala University, Uppsala, Sweden; Department of Public Health and Caring Sciences, Uppsala University, Uppsala, Sweden; Department of Women’s and Children’s Health, Uppsala University, Uppsala, Sweden; Department of Learning, Informatics, Management and Ethics, Karolinska Institute, Stockholm, Sweden

## Abstract

Inequalities in early childhood health persist across high-income countries, and structured home visiting is a principal policy response. Because such programmes are jointly produced by health and social services financed from separate budgets, the authority that pays is often not the authority that saves. We estimated the annual public-sector cost of scaling Sweden’s extended home visiting programme nationally, and how it distributes between regional healthcare and municipal social services. A scenario-based costing analysis from a public-payer perspective. National registers gave an annual target population of 14 349 families. Unit costs came from micro-costing four pilot sites in disadvantaged areas in 2021. Three scenarios were modelled: a National Standard base case, a Facility-Based Adaptation, and an Enhanced Pilot. Costs are in 2023 Euros. Unit cost per family ranged from EUR 512 (Facility-Based) to EUR 1128 (Enhanced Pilot). Scaling the National Standard model would cost EUR 8.31 million annually, the Enhanced Pilot EUR 11.33 million. Regional healthcare carried 70%–76% of costs under every delivery model, because coordination, supervision, and training are overwhelmingly nurse and midwife time. Implementation choices change total cost but not its distribution between payers. Moving contacts from the home to a facility halves the cost and removes the setting through which the programme is theorized to reach marginalized families, without rebalancing the burden. Because the asymmetry follows from how the programme is staffed, scale-up requires explicit cross-sectoral financing agreements wherever health and social care budgets are held separately.

## Introduction

Socioeconomic inequalities in early childhood health persist across high-income countries, including those with universal, tax-funded welfare systems [1]. Structured home visiting, repeated scheduled visits to families with infants by a nurse or equivalent professional, has become the principal policy instrument for narrowing these gaps, and adaptations now operate across Europe and North America. Intensive, individually targeted models such as the Nurse-Family Partnership in the United States and its licensed English adaptation, the Family Nurse Partnership, offer up to 64 visits from early pregnancy to the child’s second birthday, yet recent randomized trials of both show limited effects on their primary outcomes [2, 3]. European adaptations (ProKind in Germany, Preparing for Life in Ireland) likewise report benefits on some outcomes and subgroups only [4, 5]. Lower-intensity models layered onto existing universal child health services have consequently attracted policy interest, but their delivery costs at scale are poorly documented.

Whatever their intensity, these programmes are jointly produced by health and social services, which in most countries are financed from separate budgets, held by separate authorities, and realize returns over different time horizons. Cost and saving therefore fall to different authorities, a recognized obstacle to intersectoral prevention in settings as different as Sweden, Quebec, and South Australia [6]. Resource constraint also erodes delivery: a synthesis of four English health-visiting studies found proportionate-universalist services drift towards shorter, clinic-based contacts when capacity is squeezed, with the families in greatest need the first to lose contact [7]. Economic evaluations of home visiting have concentrated on societal return on investment and paid little attention to how costs fall across payers.

Sweden provides an instructive case because the division of responsibility is unusually sharp. Healthcare is financed by 21 Regions and social services by 290 Municipalities, each with independent taxation powers and separate budgets [8]. Since 2014, the national child health programme has included two universal home visits, at birth and at 8 months, alongside clinic-based health checks. The ‘Rinkeby model’, introduced in a disadvantaged Stockholm district in 2013, added five further home visits delivered jointly by a child health care (CHC) nurse employed by the Region and a social worker employed by the Municipality. Evaluations reported 94% participation, higher measles, mumps, and rubella vaccination coverage and lower inpatient care use at 3-year follow-up [9]; gains in parental health literacy [10, 11]; and increased parental trust in social services, the mechanism through which the intervention is expected to reduce inequality [12–14]. The Commission for Equity in Health recommended national scale-up on that basis, valuing lifelong exclusion for one individual at EUR 0.47 million [15]. Ordinance (2025:74) now codifies a permanent state grant requiring funded programmes to be jointly delivered and targeted to areas with high exclusion [16]. The 2026 grant went to 18 of Sweden’s 21 Regions and 158 of its 290 Municipalities, against 17 and 106 the previous year [17].

Scale-up nonetheless faces this fiscal problem in a particularly explicit form. The Region finances nurses and captures short-term health savings; the Municipality finances social workers and realizes savings, such as reduced social assistance, only over a much longer horizon [8]. State grants cover direct salary costs but not the asymmetric burden of coordination, travel, and administration. Decision-makers, therefore, lack an estimate of the national resources required and of how those resources fall across the two budget holders. This study addresses that gap by estimating (i) the total annual programme cost of national roll-out and (ii) its distribution between regional healthcare and municipal social services under alternative implementation models.

## Methods

### Study design and analytic framework

A national scale-up costing analysis was conducted to estimate the annual financial implications of scaling the extended home visiting programme ‘Together for a Safe Start’ to all eligible socioeconomically disadvantaged areas in Sweden. The intervention extends the national child health programme by adding six collaborative home visits conducted jointly by a CHC nurse and a social worker during the child’s first 15 months. The expanded pilot protocol additionally included two supplemental sessions: a pre-birth visit at the maternal healthcare centre and a follow-up visit at the child healthcare centre when the child turns two [18].

Relationship to the routine programme. Ordinance (2025:74) defines the extended programme as five home visits additional to the ordinary first home visit already in the national child health programme [16]. Ordinary visits are nurse-delivered; joint attendance by a social worker is specific to the extended programme. The estimates reported here are the gross cost of delivering the extended programme’s contacts (six home visits, plus two supplementary facility sessions in the Enhanced Pilot model) and, therefore, include the newborn visit that would have occurred under the routine programme anyway. They consequently overstate the true incremental cost by approximately one to two nurse-delivered home visits per family. Remaining routine CHC is excluded and, being common to all scenarios, does not affect comparisons between them.

The analysis took a public-payer perspective, capturing costs incurred by regional healthcare authorities (nurses, midwives, dental hygienists) and municipal social services (social workers, family support). Parental time lost attending visits was recorded in the micro-costing but is excluded here, since it falls on neither public payer; it accounted for 4%–8% of each site’s total. A 1-year horizon was used, without discounting. Costs were collected in 2021 Swedish Krona and are reported in 2023 Euros at purchasing power parity; Krona equivalents are derived from the 2021 cost data adjusted by the consumer price index rather than back-converted (Appendix S2).

The analytic framework follows the scaling-up principles of Milat *et al*. [19], which identify costs and contextual factors as critical determinants of successful expansion. National roll-out was conceptualized as a function of core intervention components (joint nurse and social worker visits to first-time parents in high-need areas), local implementation adaptations (notably home-based vs. facility-based delivery) and their associated unit costs. This was operationalized as two archetypes from the pilot data: a home-based intensive model and a resource-constrained facility-based adaptation. The analysis is reported in accordance with the CHEERS 2022 statement.

### Data sources and unit cost estimation

Unit costs were derived from a micro-costing study of the pilot implementation at four diverse sites in socioeconomically disadvantaged areas of Sweden during 2021, using a hybrid approach combining top-down (overhead, coordination) and bottom-up (personnel time, materials) methods. The micro-costing captured resource use specific to the target population, including professional interpretation. A site-specific cost per enrolled family was calculated to reflect structural variation in staffing configuration, visit location and protocol fidelity, using an established cost converter [20]. The study was approved by the Swedish Ethical Review Authority (Ref. 2022-00783-01) and conducted in line with the Declaration of Helsinki; informed consent was obtained from all participants.

### Target population and eligibility

The annual target population was estimated from linked Swedish national registers, using the analytical sample of a parallel study of the same programme [21]. It comprised all first-born children resident in Demographic Statistical Areas with a Care Need Index score indicating high socioeconomic disadvantage. Data covering 2012–22 identified 157 844 eligible children over 11 years, giving a modelled annual cohort of 14 349 families.

### Implementation scenarios

Because regions adapt programme intensity to local resources and service structures, costs were modelled across three scenarios representing delivery archetypes observed during the pilot:

Scenario 1—National Standard (base case): adherence to the core protocol of six home visits without additional facility-based sessions. It uses the unit costs of the high-fidelity site minus the costs of non-core visits, and is the primary comparator for national scale-up.

Scenario 2—Facility-Based Adaptation (low fidelity): modelled on pilot Site D, a lower-resource adaptation characterized by co-located services in a family centre, reduced travel, and more contacts delivered at the facility than in the home. Because most contacts do not take place in the home, it is more accurately described as a facility-based adaptation than a home visiting programme in the strict sense; the implications are examined in the Discussion.

Scenario 3—Enhanced Pilot (high fidelity): modelled on pilot Site C, this is a resource-intensive approach characterized by strict adherence to the extended protocol including pregnancy and 2-year visits, dispersed service locations requiring travel time, and high coordination costs.

### Costing and sectoral distribution

The gross annual cost was calculated for each scenario as the product of eligible population, uptake rate and model-specific unit cost. A 70% baseline uptake was applied, consistent with average enrolment in pilot administrative logs.

To inform cross-sectoral financing agreements, costs were stratified by payer. Each cost block was allocated by the professions named in it: child health nurse, midwife, and dental hygienist time to the Region, social worker and social-service manager time to the Municipality. Visit delivery is itemized by profession in the micro-costing and could be allocated exactly; set-up, coordination, supervision, and materials were apportioned by the wage-weighted staff composition described for each. One input, the employing authority of a manager credited with coordination time at one site, is not stated and was allocated equally, with alternatives tested in sensitivity analysis. The full derivation is in Appendix S4.

Deterministic sensitivity analyses varied the uptake rate (60%–90%), the regional funding share (60%–80%) around the derived value, and the eligible population, the denominator of every national estimate, on which regions apply differing criteria. Appendix S6 reports the results.

## Results

### Site-level unit costs and implementation variation

The estimated unit cost per enrolled family ranged from EUR 512 in the lowest-cost model (Site D) to EUR 1128 in the most resource-intensive model (Site C). This two-fold difference was driven primarily by structural implementation choices. The Facility-Based Adaptation (Site D) used co-located services and delivered more contacts at the healthcare centre (113 facility contacts vs. 61 home visits), reducing travel and coordination overhead. The Enhanced Pilot (Site C) operated with dispersed services and adhered strictly to the home-visiting protocol (121 home visits vs. 48 facility contacts), requiring higher expenditure on travel time and cross-sectoral coordination.

### National costing scenarios

Based on an annual eligible cohort of 14 349 families, national costs were modelled across the three scenarios (Table 1). In the National Standard scenario, assuming 70% uptake, the total annual cost of scale-up was estimated at EUR 8.31 million. The choice of implementation model altered this substantially: the Facility-Based Adaptation would cost EUR 5.14 million annually, while the Enhanced Pilot protocol would raise the annual cost to EUR 11.33 million.

**Table 1:** Estimated annual national cost of scaling the extended home visiting programme.^a^

Scenario	Model	Unit cost per family (EUR)	Total annual cost (million EUR)	Regional healthcare share (million EUR)	Municipal social services share (million EUR)	Regional share (%)
1	National Standard (base case)	827	8.31	5.81	2.50	70.0
2	Facility-Based Adaptation (low fidelity)	512	5.14	3.86	1.28	75.0
3	Enhanced Pilot (high fidelity)	1128	11.33	8.58	2.75	75.7

a Estimates assume an annual eligible population of 14 349 families and 70% uptake. Payer shares are derived from the professional mix named in each cost block of the micro-costing (Appendix S4). Costs are in 2023 Euros converted at purchasing power parity, and exclude parental time, which falls on neither public payer.

### Sectoral cost distribution

Under the National Standard scenario, the annual cost was distributed as EUR 5.81 million to the Regions and EUR 2.50 million to the Municipalities, a regional share of 70%. It was 75% in the Facility-Based Adaptation and 76% in the Enhanced Pilot, so the Region carried between 70% and 76% of costs under every model examined.

Visit delivery is close to evenly shared, as the protocol intends: at three of the four sites, it split between 50% and 62% regional. Coordination, scheduling, supervision, initial training, and written materials are almost entirely nurse and midwife time, and, therefore, regional; the municipal contribution is largely confined to the social worker’s presence at visits. Varying the one ambiguous allocation moved the National Standard share only between 68% and 72%, leaving the other scenarios unchanged.

### Sensitivity analysis

Total cost was highly responsive to uptake. In the National Standard scenario, increasing uptake from 70% to 90% raised the total annual cost from EUR 8.31 million to EUR 10.68 million; at 60% uptake the cost fell to EUR 7.12 million. Varying the regional share from 60% to 80% moved the burden on the Regions between EUR 4.98 and EUR 6.65 million, with the municipal burden moving correspondingly (Appendix S5).

## Discussion

Scaling the intervention to all 14 349 eligible families in Sweden’s socioeconomically disadvantaged areas would require an annual investment of EUR 5.1–11.3 million, depending on the implementation model adopted. Regional healthcare carries 70%–76% of that total under every model examined.

### Comparability with international programmes

The cost per visit reported here is the cost per delivered extended-programme contact: the unit cost per enrolled family in the National Standard model (EUR 827) divided by the mean number of programme visits families actually received (4.2), giving approximately EUR 197. It refers to the extended programme’s visits, the large majority of which are home visits, and not to a routine clinic-based child health visit.

Comparison with international figures requires care, because the programmes differ in dose, staffing, and target group. The Family Nurse Partnership in England and the Nurse-Family Partnership in the United States are intensive, individually targeted, standalone services offering up to 64 visits from early pregnancy to the child’s second birthday, delivered by a single specially trained nurse to young or low-income first-time mothers [2, 3]. The Swedish programme is far lower in dose (six joint visits over 15 months), but each contact involves two professionals employed by two different authorities, and it is layered on a universal service that already reaches virtually all children. Its higher cost per visit than the integrated English implementation (EUR 55), therefore, reflects two staff per contact and Swedish wage levels, while its much lower cost per family than United States models (EUR 328 per visit) reflects the smaller number of visits [22, 23]. Cost per family for a defined programme is the comparable quantity; by that measure, the Swedish model sits at the low end of the international range.

The high-fidelity scenario, ∼103 MSEK, sits below the 145 MSEK distributed nationally in 2025 under the state grant [17]. That margin depends on who is eligible. The grant covers high-fidelity delivery up to about 20 000 eligible families a year, approximately the point at which eligibility would widen from first-time parents to all children in the most disadvantaged fifth of neighbourhoods. Within that boundary, the binding constraint on scale-up is how the money is divided between payers.

### Delivery setting and the trade-off between fidelity and efficiency

The two-fold cost difference between scenarios is driven by where the contact happens. The Facility-Based Adaptation reduces the annual national cost to EUR 5.14 million, roughly half the Enhanced Pilot, but achieves this by moving the majority of contacts out of the home. Whether such a model remains a home visiting programme determines what policymakers should expect the money to buy.

The three delivery modes are not interchangeable. The routine child health programme provides universal clinic-based surveillance with two home visits and reaches nearly all children efficiently, but is not designed to address the social determinants that generate the inequalities of concern. Family centres, which co-locate CHC, social services, open pre-school, and midwifery, lower the threshold for reaching several services in one place, enable informal peer contact between parents, and reduce staff travel and coordination time, the main source of the savings in Scenario 2. They cannot reproduce the mechanism attributed to the home visit. Swedish nurses and social workers describe the home as giving access to the family’s actual living conditions, allowing both parents and siblings to be present, shifting the balance of authority towards the family, and reaching families who do not attend clinic appointments [24]. Qualitative evidence links the home setting specifically to building trust with social services [12, 13], and delivery disparities track the same gradient [14].

The same shift towards facility-based contact under resource pressure is documented in England, where proportionate-universalist health visiting moves to shorter, clinic-based contacts, with the most disadvantaged families disproportionately affected [7]. Scenario 2 should, therefore, be read as a lower bound on cost, carrying a correspondingly lower expectation of equity impact. Its low-cost profile also depends on co-located infrastructure that is not universally available.

### Fragmented financing as a general problem

A programme designed as a partnership between two authorities is not, on these data, financed as one. Regional healthcare carries 70% of costs under the National Standard model, 75% under the Facility-Based Adaptation, and 76% under the Enhanced Pilot. The imbalance is, therefore, not produced by the choice of delivery location, and cannot be corrected by changing it. It follows instead from how the programme is staffed: the joint visit is genuinely shared, but the infrastructure that makes the visit possible (coordination, scheduling, supervision, initial training, and written materials) is nurse and midwife time and falls to the Region. The Municipality contributes the social worker’s presence at visits and little else.

A region under budget pressure might expect that moving contacts to a family centre would both save money and distribute the cost more evenly. The facility-based model is cheaper and simultaneously more regionally skewed, because facility contacts substitute nurse time for travel without adding municipal input. A substantial share of the returns, reduced social assistance and reduced child protection involvement, accrues to the Municipality over a longer horizon.

Wherever preventive services are jointly produced across separately financed health and social care budgets, the greater part of the work and the greater part of the returns fall to different budgets, and the predictable equilibrium is under-provision or drift towards the cheaper model [6, 7]. Such programmes, therefore, cannot be costed at the level of the intervention alone: unless the overheads surrounding delivery are attributed to a payer, the financing requirement is misstated. Payer-disaggregated costing is a precondition for the pooled budgets, transfer payments or ring-fenced grants that could correct the imbalance.

A further structural cost is professional interpretation. More than half of residents in these areas are foreign-born, and interpretation is a standing requirement of delivery; capping it would compromise the programme’s capacity to reach the population it was designed for.

### Strengths and limitations

We have not located an earlier estimate of national scale-up costs and payer-specific fiscal distribution for an extended home visiting programme. The study’s principal strength is micro-costing data from four sites that implemented the programme differently, rather than protocol estimates, with an established scaling-up framework used to define the archetypes.

Several limitations apply. The analysis relies partly on manager-reported time estimates for non-clinical tasks, which may introduce recall bias. The payer allocation is exact for visit delivery but rests on described staff composition for coordination, supervision and set-up, where one manager’s employing authority is not recorded. Pilot delivery took place during the COVID-19 pandemic, which both reduced visit frequency and shifted some contacts away from the home. The eligible population is first-born children in high-need areas, matching the programme’s design; regions apply differing criteria, so Appendix S6 reports the population at which each scenario exhausts the grant. As a cost analysis, the study does not calculate net budget impact, societal benefits, or a net incremental cost relative to the routine programme. Travel costs were modelled implicitly and may be understated for sparsely populated regions, as the pilot sites were urban or semi-urban. Finally, the sites were selected for readiness and cannot be assumed representative of all areas covered at national scale.

## Conclusion

Scaling the extended home visiting programme is financially feasible, and the state grant already exceeds what national high-fidelity delivery would require. Policymakers can minimize cost through facility-based adaptations, or maximize fidelity to the home-visiting protocol at roughly double the investment; because the intervention’s benefits are theorized to depend on reaching families in their own environment, financing should prioritize the home-based standard. Regional healthcare carries 70%–76% of costs whichever model is adopted, because the coordination and supervision surrounding the joint visit are almost entirely health service time. Sustaining the programme therefore depends on an explicit financing agreement between the two authorities, and the same requirement arises in any system where prevention is paid for from one budget and its returns accrue to another.

## Supplementary Material

ckag174_Supplementary_Data

## Data Availability

The aggregated cost data generated during the study are available in the Supplementary Information. Raw micro-costing logs are not publicly available due to privacy restrictions but are available from the corresponding author on reasonable request. The R code used for the analyses is available from the corresponding author on request. Key points Scaling an extended home visiting programme nationally in Sweden would cost EUR 5.1–11.3 million annually, varying two-fold with the delivery model chosen and in all three scenarios below the state grant already allocated. Regional healthcare carries 70%–76% of the cost under every delivery model, because programme coordination, supervision and training are almost entirely nurse and midwife time. Moving contacts from the home to a co-located family centre halves the cost but makes the payer imbalance greater, while removing the setting through which the programme is theorized to reach marginalized families. Cross-sectoral prevention cannot be costed at the level of the intervention alone; unless surrounding overheads are attributed to a payer, the financing requirement is misstated.
