# Supplementary material for: Scaling an equity-oriented extended home visiting programme in Sweden: costs and cross-sectoral fiscal distribution
Source: Eur J Public Health. 2026 Sep 25;36(5):ckag174. doi: 10.1093/eurpub/ckag174 (PMC13614793; doi:10.1093/eurpub/ckag174)
Supplement: Supplementary file 1 [file ckag174_supplementary_data.zip › ejph-2025-12-om-1084-File002.docx]

**Supplementary material**

Scaling an equity-oriented extended home visiting programme in Sweden: costs and cross-sectoral fiscal distribution

**Appendix S1. Input parameters**

Unit costs and resource-use inputs are taken from a micro-costing of the four pilot sites during 2021. Costs are given in 2021 prices, the year of data collection; the conversion to 2023 price levels and to Euros is described in Appendix S2.

*Table S1a. Unit costs of resources (2021 SEK)*

| **Resource** | **Unit cost (2021 SEK)** | **Unit** | **Source** |
| --- | --- | --- | --- |
| Child health care nurse (Region) | 252.50 | per hour | Statistics Sweden |
| Social worker (Municipality) | 225.63 | per hour | Statistics Sweden |
| Midwife (Region) | 262.50 | per hour | Statistics Sweden |
| Dental hygienist (Region) | 211.88 | per hour | Statistics Sweden |
| Interpreter | 226.88 | per hour | Statistics Sweden |
| Parental time (production loss) | 182.10 | per hour | Statistics Sweden |
| Written material | 42.00 | per folder | National Board of Health and Welfare |

*Table S1b. Delivered contacts and mean contact duration, by site (2021)*

| **Site** | **Total contacts** | **Home visits** | **Facility visits** | **Other or online** | **Mean home visit (min)** | **Mean facility visit (min)** |
| --- | --- | --- | --- | --- | --- | --- |
| Site A | 193 | 121 | 71 | 2 | 82 | 73 |
| Site B | 140 | 118 | 19 | 3 | 75 | 58 |
| Site C | 185 | 121 | 48 | 16 | 70 | 44 |
| Site D | 175 | 61 | 113 | 1 | 59 | 45 |

Note. The National Standard scenario is modelled on the mean home-visit duration of the two high-fidelity sites, A and C. The Facility-Based Adaptation is modelled on the facility-visit duration observed at Site D.

**Appendix S2. Price year and currency conversion**

Costs were collected in Swedish Krona at 2021 prices. Two conversions are applied, and because they serve different purposes they use different rates.

Euros, for international comparison. Site costs were adjusted to 2023 prices and converted to Euros using the CCEMG-EPPI-Centre cost converter, which applies purchasing power parities rather than market exchange rates. The implied rate across the four sites is 9.11 to 9.48 SEK per Euro. Purchasing power parity is the appropriate basis for comparing resource use across health systems, and the Euro figures in the manuscript are on this basis.

Swedish Krona, for domestic budget context. Krona figures are built from the 2021 cost data and inflated to 2023 prices using the Swedish Consumer Price Index (Statistics Sweden: 8.4 per cent in 2022 and 8.5 per cent in 2023, a cumulative factor of 1.176). They are not converted from the Euro figures, since purchasing power parities and market exchange rates differ and applying one in each direction would not return the original values.

*Table S2. Site-level cost build-up*

| **Site** | **Total cost (2021 SEK)** | **Families** | **Per family (2021 SEK)** | **Per family (2023 SEK)** | **Per family (2023 EUR, PPP)** | **Implied SEK per EUR** |
| --- | --- | --- | --- | --- | --- | --- |
| Site A | 380 099 | 49 | 7 757 | 9 123 | 962 | 9.48 |
| Site B | 293 429 | 45 | 6 521 | 7 669 | 830 | 9.24 |
| Site C | 402 250 | 44 | 9 142 | 10 752 | 1 180 | 9.11 |
| Site D | 214 248 | 48 | 4 464 | 5 250 | 556 | 9.44 |

**Appendix S3. Scenario unit costs**

Scenario 1 (National Standard) is the simple mean across the four pilot sites; Scenario 2 (Facility-Based Adaptation) is Site D; Scenario 3 (Enhanced Pilot) is Site C. Each is reported gross and on a public-payer basis. The gross figure is the whole cost recorded in the micro-costing, which took a limited societal perspective and included parental time lost in attending visits. The public-payer figure excludes parental time, since it falls on neither the Region nor the Municipality, and is the basis used in Table 1 of the manuscript.

*Table S3. Scenario unit costs, gross and public-payer basis*

| **Scenario** | **Basis** | **Gross, incl. parental time (EUR)** | **Public payer only (EUR)** | **Public payer only (2023 SEK)** |
| --- | --- | --- | --- | --- |
| 1. National Standard | Mean of four sites | 882 | 827 | 7 684 |
| 2. Facility-Based Adaptation | Site D | 556 | 512 | 4 833 |
| 3. Enhanced Pilot | Site C | 1 180 | 1 128 | 10 277 |

Note. The families-weighted mean across the four sites is EUR 877 gross, against the simple mean of EUR 882 used here.

**Appendix S4. Distribution of costs between payers**

Each cost block in the micro-costing was allocated according to the professions named in that block. Time for child health nurses, midwives and dental hygienists is a regional cost; time for social workers and social-service managers is a municipal cost. Parental production loss falls on neither public payer and is reported separately.

Hourly wage rates are those in Table S1a. An implied working year of 1 920 hours is used for staff costed as a proportion of full time. This is confirmed by the Site B coordination entry, where one nurse at 15 per cent gives 0.15 x 1 920 x 252.50 = 72 720 SEK, the figure recorded in the micro-costing.

*Table S4a. Allocation of costs by site (2021 SEK)*

| **Site** | **Region** | **Municipality** | **Parental time** | **Regional share of public cost** |
| --- | --- | --- | --- | --- |
| Site A | 213 506 | 136 637 | 29 965 | 61.0% |
| Site B | 188 197 | 88 199 | 17 033 | 68.1% |
| Site C | 291 169 | 93 290 | 17 791 | 75.7% |
| Site D | 147 999 | 49 232 | 17 017 | 75.0% |

*Table S4b. Regional share of costs, visit delivery alone and all cost blocks*

| **Site** | **Visit-delivery costs only** | **All cost blocks** |
| --- | --- | --- |
| Site A | 60.9% | 61.0% |
| Site B | 49.6% | 68.1% |
| Site C | 62.3% | 75.7% |
| Site D | 70.5% | 75.0% |

The regional share of public costs is 61 to 76 per cent across the four sites. Visit delivery alone splits more evenly, between 50 and 62 per cent regional at three of the four. Programme coordination, scheduling, supervision, initial training and written material are almost entirely nurse and midwife time, while the municipal contribution is largely the social worker's presence at visits.

One input is not recorded in the micro-costing: the employing authority of the manager credited with 10 per cent coordination time at Site A. Allocating that time wholly to the Municipality or wholly to the Region moves the Scenario 1 share between 68.2 and 71.7 per cent, and leaves Scenarios 2 and 3 unchanged.

Applying these shares gives the distribution in Table S4c. The regional share is high under every delivery model: a facility-based adaptation reduces total cost without distributing it more evenly, so the asymmetry follows from how the programme is staffed.

*Table S4c. Scenario costs by payer, public-payer perspective*

| **Scenario** | **Unit cost per family (EUR)** | **Total annual cost (million EUR)** | **Regional healthcare (million EUR)** | **Municipal social services (million EUR)** | **Regional share** |
| --- | --- | --- | --- | --- | --- |
| 1. National Standard | 827 | 8.31 | 5.81 | 2.50 | 70.0% |
| 2. Facility-Based Adaptation | 512 | 5.14 | 3.86 | 1.28 | 75.0% |
| 3. Enhanced Pilot | 1 128 | 11.33 | 8.58 | 2.75 | 75.7% |

Note. Totals assume 10 044 families, being 14 349 eligible at 70 per cent uptake. Parental time is excluded, as in Appendix S3.

**Appendix S5. Sensitivity analysis**

A two-way deterministic sensitivity analysis was performed on the National Standard scenario, varying programme uptake from 60 to 90 per cent of the eligible population and the regional share of total cost from 60 to 80 per cent around the derived value of 70 per cent, with the Municipality covering the remainder.

*Table S5. Annual national cost under varying uptake and payer split*

| **Uptake** | **Regional share** | **Total (million EUR)** | **Region (million EUR)** | **Municipality (million EUR)** | **Total (MSEK)** |
| --- | --- | --- | --- | --- | --- |
| 60% | 60% | 7.12 | 4.27 | 2.85 | 66.2 |
|  | 70% (base) | 7.12 | 4.98 | 2.14 |  |
|  | 80% | 7.12 | 5.70 | 1.42 |  |
| 70% (base) | 60% | 8.31 | 4.98 | 3.32 | 77.2 |
|  | 70% (base) | 8.31 | 5.81 | 2.49 |  |
|  | 80% | 8.31 | 6.64 | 1.66 |  |
| 80% | 60% | 9.49 | 5.70 | 3.80 | 88.2 |
|  | 70% (base) | 9.49 | 6.64 | 2.85 |  |
|  | 80% | 9.49 | 7.59 | 1.90 |  |
| 90% | 60% | 10.68 | 6.41 | 4.27 | 99.2 |
|  | 70% (base) | 10.68 | 7.48 | 3.20 |  |
|  | 80% | 10.68 | 8.54 | 2.14 |  |

Note. Based on an annual eligible population of 14 349 families and a public-payer unit cost of EUR 827, equivalent to SEK 7 684. Krona totals are built from the 2021 cost data inflated to 2023 prices, as described in Appendix S2.

**Appendix S6. Sensitivity to the eligible population**

Every national estimate in this study is the product of an eligible population, an uptake rate and a unit cost. The eligible population is an annual cohort of 14 349 families, obtained as 157 844 first-born children resident in high-need areas over eleven years of register data. Because the state grant is compared against these totals, and because regions apply differing eligibility criteria in practice, this appendix reports the cost across a range of definitions and the population at which the grant is exhausted.

*Table S6a. Annual national cost by eligible population, at 70% uptake*

| **Eligible families, annual** | **Basis** | **Scenario 1** | **Scenario 2** | **Scenario 3** |
| --- | --- | --- | --- | --- |
| 7 763 | First-born, most disadvantaged fifth | 42 | 26 | 56 |
| 14 349 | First-born, high-need areas (base case) | 77 | 49 | 103 |
| 16 593 | First-born, two most disadvantaged fifths | 89 | 56 | 119 |
| 20 044 | All children, most disadvantaged fifth | 108 | 68 | 144 |
| 40 203 | All children, two most disadvantaged fifths | 216 | 136 | 289 |

Note. Costs are million SEK at 2023 prices. Reference counts for the alternative definitions are from the national cohort of children born 2012 to 2022 with parity and a neighbourhood Care Need Index recorded (n = 1 111 622), annualised over eleven years. The base case is 33% of all first-born children and 14% of all children born in the period. The first-born restriction follows the programme's design, and removing it multiplies the cohort by about 2.4.

*Table S6b. Population at which the 2025 state grant of 145 MSEK is exhausted*

| **Scenario** | **Eligible families, annual** | **Relative to base case** |
| --- | --- | --- |
| 1 National Standard | 26 956 | 1.88 |
| 2 Facility-Based | 42 862 | 2.99 |
| 3 Enhanced Pilot | 20 156 | 1.40 |

Note. The grant covers each scenario up to the population shown. For high-fidelity delivery this is approximately the register count of all children in the most disadvantaged fifth of areas (20 044), that is, the point at which eligibility would widen from first-time parents to every child in those areas.

A costing built from reimbursement tariffs would overstate the regional cost. At approximately 1 800 SEK per nurse visit and the six visits the protocol specifies, a region would budget 10 800 SEK per family, whereas the regional cost observed here was close to half that, because delivered visit frequency was 4.2 against the six planned.
