# Supplementary material for: Scaling an equity-oriented extended home visiting programme in Sweden: costs and cross-sectoral fiscal distribution
Source: Eur J Public Health. 2026 Sep 25;36(5):ckag174. doi: 10.1093/eurpub/ckag174 (PMC13614793; doi:10.1093/eurpub/ckag174)
Supplement: Supplementary file 1 [file ckag174_supplementary_data.zip › ejph-2025-12-om-1084-File003.docx]

**CHEERS 2022 Checklist**

Scaling an equity-oriented extended home visiting programme in Sweden: costs and cross-sectoral fiscal distribution. Manuscript EJPH-2025-12-OM-1084.R1. Section names refer to the headings used in the manuscript, which are not numbered.

| **Item** | **Section/Topic** | **Recommendation** | **Reported on Section** |
| --- | --- | --- | --- |
| **Title and Abstract** |  |  |  |
| 1 | Title | Identify the study as an economic evaluation (or cost analysis). | Title |
| 2 | Abstract | Provide a structured summary. | Abstract |
| **Introduction** |  |  |  |
| 3 | Background and Objectives | Context and study objective (PICO). | Background (final paragraph) |
| **Methods** |  |  |  |
| 4 | Health Economic Analysis Plan | Indicate if a plan was developed. | Methods - Study design and analytic framework |
| 5 | Study Population | Describe characteristics of the study population. | Methods - Target population and eligibility |
| 6 | Setting and Location | Describe setting(s) and location(s). | Methods - Data sources and unit cost estimation (four pilot sites, 2021) |
| 7 | Comparators | Describe interventions or strategies being compared. | Methods - Implementation scenarios |
| 8 | Perspective | State the perspective of the analysis. | Methods - Study design and analytic framework (public-payer perspective) |
| 9 | Time Horizon | State the time horizon(s). | Methods - Study design and analytic framework (one-year horizon) |
| 10 | Discounting | Report discount rate(s). | Methods - Study design and analytic framework (not applied) |
| 11 | Selection of Outcomes | Describe outcomes used. | Methods - Study design and analytic framework (total cost and its distribution between payers) |
| 12 | Measurement of Outcomes | Describe how outcomes were measured. | Not applicable (cost analysis; no health outcomes estimated) |
| 13 | Valuation of Outcomes | Describe how outcomes were valued. | Not applicable (cost analysis; no health outcomes estimated) |
| 14 | Measurement and Valuation of Resources and Costs | Describe how costs were estimated. | Methods - Data sources and unit cost estimation; Appendix S1 |
| 15 | Currency, Price Date, and Conversion | Report dates and currency adjustments. | Methods - Study design and analytic framework (2021 SEK, reported in 2023 EUR at PPP); Appendix S2 |
| 16 | Choice of Model | Describe modelling approach. | Methods - Study design and analytic framework (scaling-up framework); Implementation scenarios |
| 17 | Assumptions | Describe structural assumptions. | Methods - Implementation scenarios; Costing and sectoral distribution; Appendix S4 |
| 18 | Analytic Methods | Describe methods to characterize uncertainty. | Methods - Costing and sectoral distribution (final paragraph); Appendices S5 and S6 |
| **Results** |  |  |  |
| 19 | Study Parameters | Report values for all parameters. | Results - Site-level unit costs and implementation variation; Appendices S1 and S3 |
| 20 | Incremental Costs and Outcomes | Report mean values/differences. | Results - National costing scenarios; Table 1 |
| 21 | Characterizing Uncertainty | Report effect of sampling uncertainty. | Results - Sensitivity analysis; Appendices S5 and S6. Deterministic one-way and two-way analyses only; no probabilistic sensitivity analysis was undertaken |
| 22 | Heterogeneity | Report differences between subgroups. | Results - Site-level unit costs and implementation variation (variation across the four sites); Appendix S2 |
| **Discussion** |  |  |  |
| 23 | Discussion of Findings | Summarize key findings. | Discussion (opening paragraph); Comparability with international programmes |
| 24 | Effect of Uncertainty | Discuss key drivers of results. | Discussion - Delivery setting and the trade-off between fidelity and efficiency; Fragmented financing as a general problem |
| 25 | Study Findings, Limitations, Generalizability | Discuss generalizability and limitations. | Discussion - Strengths and limitations |
| 26 | Engagement with Patients | Describe patient engagement. | Not applicable (no patient or public involvement in the economic design) |
| **Other** |  |  |  |
| 27 | Conflicts of Interest | Describe conflict of interest. | Statements and Declarations (Competing Interests) |
| 28 | Funding | Report source of funding. | Statements and Declarations (Funding) |
